# Predictors of long-term outcome following high-dose chemotherapy in high-risk primary breast cancer

**DOI:** 10.1038/sj.bjc.6600450

**Published:** 2002-08-01

**Authors:** G Somlo, J F Simpson, P Frankel, W Chow, L Leong, K Margolin, R Morgan, J Raschko, S Shibata, S Forman, N Kogut, M McNamara, A Molina, E Somlo, J H Doroshow

**Affiliations:** Department of Medical Oncology and Therapeutics Research, City of Hope National Medical Center, 1500 E Duarte Road, Duarte, California, CA 91010-3000, USA; Department of Biostatistics, City of Hope National Medical Center, 1500 E Duarte Road, Duarte, California, CA 91010-3000, USA; Department of Hematology and Bone Marrow Transplantation, City of Hope National Medical Center, 1500 E Duarte Road, Duarte, California, CA 91010-3000, USA; Division of Anatomic Pathology, Vanderbilt University Medical Center, Nashville, Tennessee, USA

**Keywords:** high-risk breast cancer, high-dose therapy, risk factors

## Abstract

We report on a predictive model of long-term outcome in 114 high-risk breast cancer patients treated with high-dose chemotherapy between 1989 and 1994. Paraffin-blocks from 90 of the 114 primaries were assessed for the presence of five risk factors: grade, mitotic index, protein expression of p53, HER2/neu, and oestrogen/progesterone receptor status; we could analyse the effect of risk factors in 84 of these 90 tumours. Seven-year relapse-free and overall survival was 58% (95% confidence interval 44–74%) and 82% (95% confidence interval 71–94%) *vs* 33% (95% confidence interval 21–52%) and 41% (95% confidence interval 28–60%) for patients whose primary tumours displayed ⩾3 risk factors *vs* patients with ⩽2 risk factors. For the entire group of 168 high-risk breast cancer patients, inflammatory stage IIIB disease and involved post-mastectomy margins were associated with decreased relapse-free survival and overall survival; patients treated with non-doxorubicin containing standard adjuvant therapy experienced worse overall survival (RR, 2.08; 95% confidence interval 1.04 to 4.16; *P*=0.04), while adjuvant tamoxifen improved overall survival (RR, 0.65; 95% confidence interval 0.41–1.01; *P*=0.054). Future trial designs and patient selection for studies specific for high-risk breast cancer patients should include appropriate prognostic models. Validation of such models could come from recently completed randomised, prospective trials.

*British Journal of Cancer* (2002) **87**, 281–288. doi:10.1038/sj.bjc.6600450 www.bjcancer.com

© 2002 Cancer Research UK

## 

Approximately 50% of patients with high-risk primary breast cancer (HRBC), defined by the presence of ⩾10 involved axillary lymph nodes, T3N1, T3N2, or inflammatory primary tumours are likely to relapse within 3–5 years from the time of diagnosis (Buzdar *et al*, 1992; Bonadonna *et al*, 1995; Moon *et al*, 1997; Curcio *et al*, 1999). Improvements in relapse-free (RFS) and overall survival (OS) for these women with HRBC have been modest (Hortobagyi *et al*, 1996). Although newer agents like the taxanes have recently been evaluated as components of standard dose adjuvant therapy, patients with HRBC remain at great risk of relapse.

In 1997, we reported encouraging preliminary RFS and OS rates in a cohort of 114 patients with HRBC treated with conventional dose adjuvant therapy followed by high-dose chemotherapy (HDCT) with either doxorubicin, etoposide, and cyclophosphamide (CAVP), (Somlo *et al*, 1994a) or cisplatin, etoposide, and cyclophosphamide (CCVP) (Somlo *et al*, 1994b, 1997). Not all patients benefited equally; lack of progesterone receptor (PR) expression on the primary tumour was identified as an adverse predictor of outcome. In the current study, we set out to identify additional tumour and treatment-associated predictors of RFS and OS. Here, we report on the effect of stage, grade, mitotic index, and immunohistochemically detectable parameters (p53, HER2/neu, oestrogen and progesterone receptor status) on the outcome of a 90 patient subset of this well-defined, high-risk population of 114 patients, whose paraffin-embedded primary tumours were available for detailed analysis. Tumour and treatment-associated prognostic indicators of RFS and OS were also assessed and compared to a ‘standard’ cohort of concurrently-treated HRBC patients who had received conventional adjuvant therapy only.

## PATIENTS AND METHODS

All 114 patients participating in HDCT trials at the City of Hope and treated between 1989 and 1994 gave their written, voluntary informed consent for the study; patients with HRBC (stage II with ⩾10 involved axillary nodes, stage IIIA or B) were ⩽65-years old, with a Karnofsky performance status of ⩾80%. Patients with conventional adjuvant doxorubicin exposure of ⩾150 mg m^−2^ and/or with prior left sided chest wall radiation received CCVP (*n*=57); all others received CAVP (*n*=57). Details of the two HDCT regimens, supportive care, and treatment-related toxicities have been reported earlier (Somlo *et al*, 1997). Patients were intended to receive radiation to the primary site/chest wall and draining lymph node areas according to community standards. Patients with either oestrogen (ER) and/or PR positive breast cancer were to receive tamoxifen.

We performed a retrospective chart review of patients with HRBC who received conventional adjuvant therapy only, between the years 1989 and 1994. After obtaining approval by the Institutional Review Board of the COH, all patients with breast cancer meeting our definition of ‘high-risk’ were identified in our institutional tumour registry. Patients in the standard group underwent physical examinations and routine laboratory and radiographic evaluations according to prevailing standards of clinical practice. Of 213 HRBC cancer patients treated at our centre between 1989 and 1994 with standard chemotherapy, we could include 54 in this analysis. Reasons for exclusion from the ‘standard’ group included incomplete information on the pathology of the primary, treatment, age >65 years, or participation on a competing dose-intense protocol of doxorubicin, cyclophosphamide, and G-CSF support (Morgan *et al*, 1997); the median time from diagnosis to HDCT for patients in the HDCT group was 5.8 months; hence, ‘standard’ patients who relapsed within 6 months from diagnosis were also excluded. Characteristics of patients in the HDCT and standard treatment groups are shown in Table 1

**Table 1:**
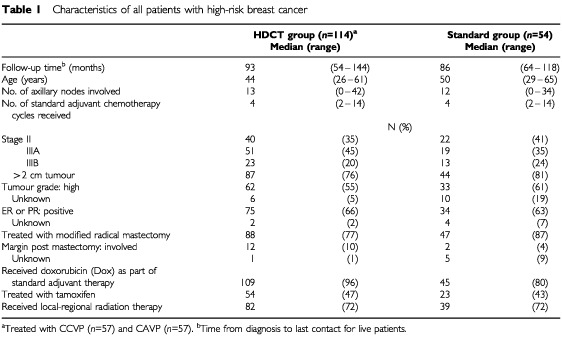
Characteristics of all patients with high-risk breast cancer

.

### Post-treatment follow-up

Following HDCT, patients underwent physical examination at least once every 4 months for the first 3 years, and every 6 months thereafter. Yearly mammograms, bone scans, and chest X-rays were performed for the first 3 years, with yearly mammograms continuing thereafter. Patients in the ‘standard’ group were followed according to prevailing clinical practice.

### Histopathologic analysis

Representative sections from all primary tumours were reviewed and analysed by a staff member of the Department of Anatomic Pathology at the City of Hope National Medical Center. Special features examined included grade, multifocality, receptor status, and vascular invasion.

More detailed analysis by the same pathologist (J Simpson) including assessment of combined histologic grade (Elston and Ellis, 1991; Dalton *et al*, 1994), mitotic index expressed as the number of mitoses per 10 high power fields (HPF), vascular invasion, and immunohistochemical features was performed on a subset of 90 of the 114 HDCT-treated patients from whom paraffin blocks of the primary tumour were available. The paraffin-embedded blocks were dewaxed in xilene and rehydrated in ethanol; 4 μm sections were placed on slides. Immunohistochemical stains were carried out following microwave epitope retrieval. Slides were stained as single batch using a Techmate 1000 immunostainer (Biotek solutions, Santa Barbara, CA, USA). Clone DO7, Novocastra, Newcastle, UK, was used at a 1 : 150 dilution to detect the presence of mutant p53; a polyclonal antibody produced by Ciba-Corning, Alameda, CA, USA, at 1 : 75 dilution, was used to detect expression of the HER2/neu protein. Ki-67 was stained utilising MiB1 by Immunotech, Mestbrook, ME, USA, at a 1 : 50 dilution; the antibodies 1D5 (Dako, Carpenteria, CA, USA; 1 : 60 dilution) and 1A6 (Novocastra, Newcastle upon Tyne, UK; dilution 1 : 10) were applied to detect ER and PR protein expression.

All primary antibody incubations were performed at room temperature for 30 min. Biotin-labelled antimouse antibodies and avidin-labelled peroxidase and diaminobenzidine detection system were used for antigen localisation (Biotek ChemMate reagents). Haematoxylin was used to counterstain the nucleus. Multi-tissue blocks were used for positive and negative controls.

Immunohistochemical scoring for p53, ER, PR, and Ki67 was considered positive based on the presence of diaminobenzipidine precipitation observed at 100×magnification. The percentage of stained nuclei was estimated, and the presence of >5% of cells showing nuclear staining was considered positive. For Her-2/neu, positive immunoreactivity was scored when diaminobenzipidine precipitation was observed as crisp membraneous staining; positive interpretation required staining of >5% of tumour cells. Intensity score was assessed using a scale of I to III.

### Statistical methods

Overall survival (OS) and relapse-free survival (RFS) were calculated from diagnosis. RFS was defined as time to any type of recurrence or death from any cause.

Univariate and multivariate Cox regression analyses were carried out to assess potential predictors (inherent to the primary tumours) and prognostic indicators (treatment-related variables) of RFS and OS such as stage, size (⩽2 cm *vs* >2 cm and ⩽5 cm *vs* >5 cm), grade (high *vs* low and intermediate), multifocality (unifocal *vs* multifocal), presence of vascular invasion (yes *vs* no), ER and PR status, number of axillary lymph nodes (⩽3, 4–9, ⩾10), Treatment-related prognostic factors included the type (doxorubicin-containing: yes *vs* no) and number of cycles of adjuvant therapy, administration of tamoxifen, and radiation treatment to the primary site (yes *vs* no). As previously reported, no significant interactions between the two HDCT regimens (CAVP *vs* CCVP) and each of the tested parameters were found (Somlo *et al*, 1997); thus, the current analysis was carried out pooling the patients treated with either of the two HDCT regimens.

For the subset of patients whose primary tumour blocks were available, mitotic activity (>3 out of 10 high power field *vs* ⩽3 out of 10 high power field), and immunohistochemical detection of nuclear staining (⩽5% *vs* >5%) for p53, ER, PR, and Ki67 (by MIB-1), and membranous staining of ⩽5% *vs* >5% for HER2/neu, were analysed. Staining intensity was also analysed grouping intensity levels of 0 and I *vs* II and III.

Standard Kaplan–Meier (Kaplan and Meier, 1958) and Cox (Cox, 1972) regression methods were applied for survival analysis using the SAS/STAT (Sas Institute, 2000) and S-Plus software. All significance testing was two-sided (log-rank statistics and Wald statistics were used in univariate and multivariate analysis, respectively).

## RESULTS

### Predictive value of histopathologic analysis in patients treated with HDCT

There was no difference in the characteristics of the entire 114 patient HDCT cohort and the 90 patient subset whose paraffin-embedded tumour blocks were procured for analysis (data not shown). Table 2

**Table 2:**
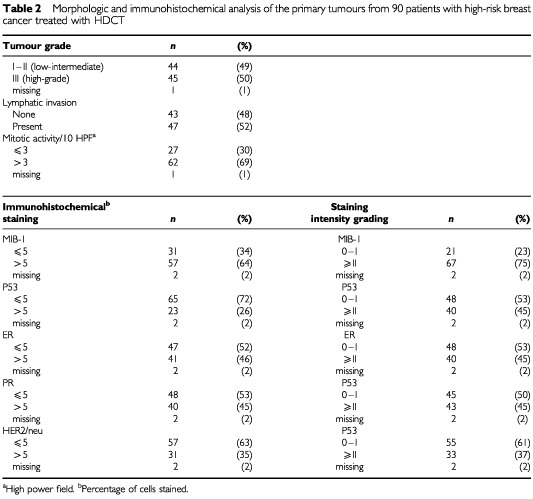
Morphologic and immunohistochemical analysis of the primary tumours from 90 patients with high-risk breast cancer treated with HDCT

describes the histopathologic findings of the tumour specimens from the 90 patients treated with HDCT. The majority of tumours (69%) were characterised by increased mitotic activity; half of the tumours were classified as high-grade and 52% contained features of vascular invasion. The proliferative marker Ki67 (as stained by MIB-1) was observed in 64% and staining intensity was high (⩾grade II) in 75% of tumour specimens. Either ER or PR positivity only, or both, was seen in 58% of the tumours. High staining intensity (⩾II) for ER or PR was observed in 45 and 48% of cases, respectively. Expression of p53 protein was seen in 26% of tumours; ⩾grade II intensity of staining was observed in 45% of tumours examined. Finally, 35% of tumours overexpressed HER2/neu; ⩾grade II staining intensity was seen in 37% of breast tumours.

Variables associated with an increased risk of relapse by univariate analysis in the 90 HDCT-treated patients included the presence of p53, overexpression of HER2/neu, and stage IIIB inflammatory disease; PR positivity and ER staining at ⩾grade II intensity were associated with a reduced risk of relapse (Table 3

**Table 3:**
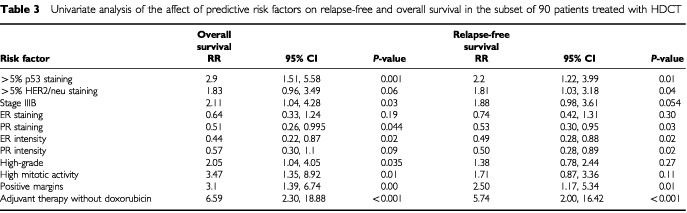
Univariate analysis of the affect of predictive risk factors on relapse-free and overall survival in the subset of 90 patients treated with HDCT

). OS was adversely affected by the presence of p53, high grade features, increased mitotic activity, and inflammatory presentation (stage IIIB), while overexpression of HER2/neu showed a trend (*P*=0.06) toward predicting shorter survival. Treatment-associated adverse prognostic indicators for both RFS and OS included involved tumour margin following mastectomy and non-doxorubicin containing standard adjuvant chemotherapy (preceding HDCT). Multivariate stepwise Cox regression analysis revealed that the presence of the p53 protein (RR, 2.06; 95% CI, 1.11–3.83; *P*=0.02) and non-doxorubicin containing adjuvant chemotherapy (RR, 5.85; 95% CI, 1.71–19.99; *P*<0.01) were predictors of increased risk for relapse, while intense PR staining was associated with lower incidence of relapse (RR, 0.55; 95% CI, 0.31–0.997; *P*=0.049). OS was adversely affected by the presence of p53, (RR, 2.15; 95% CI, 1.05–4.37; *P*=0.04), increased mitosis (RR, 3.6; 95% CI, 1.30–10.08; *P*=0.01), inflammatory presentation (RR, 2.14; 95% CI, 1.00 to 4.57; *P*=0.05), and non-doxorubicin adjuvant chemotherapy (RR, 13.4; 95% CI, 3.54–50.74; *P*<0.01).

Patients whose primary tumour blocks were analysed were assigned to favourable and unfavourable groups based on the number of adverse predictive risk features (determined to be significant by univariate analysis as shown in Table 3), to generate a scoring system. In an attempt to rely on inherent biological characteristics of the tumours, only the 86 patients treated with a doxorubicin-containing adjuvant regimen prior to HDCT therapy were to be analysed for outcome; two specimens from these patients were inadequate for p53 and HER2/staining; hence, only 84 of the 90 patients with tumour blocks were included in this subsequent analysis.

Table 4

**Table 4:**
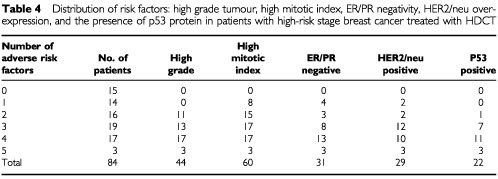
Distribution of risk factors: high grade tumour, high mitotic index, ER/PR negativity, HER2/neu overexpression, and the presence of p53 protein in patients with high-risk stage breast cancer treated with HDCT

demonstrates the distribution of the following risk factors: high mitotic index, high grade tumour, ER/PR receptor negativity, presence of p53 protein, and overexpression of HER2/neu protein. The outcome of patients with tumours demonstrating ⩽two adverse features (favourable group) was compared to those patients with ⩾three adverse features (unfavourable group). Figure 1

**Figure 1:**
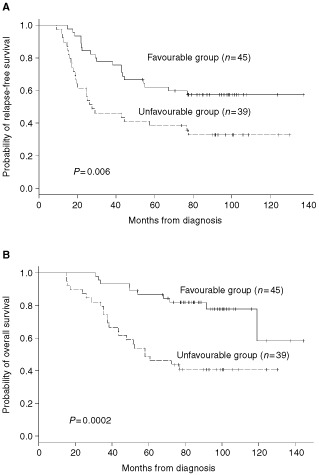
(**A**) Relapse-free survival in 84 HDCT patients displayed by the number of pathologic risk factors (⩽2 *vs* ⩾3). (**B**) Overall survival in 84 HDCT patients displayed by the number of pathologic risk factors (⩽2 *vs* ⩾3).

reveals a significantly higher risk of relapse (RR: 2.23, 95%CI 1.23–4.04; *P*=0.006) and death (RR: 3.68, 95% CI 1.75–7.75, *P*=0.0002) for patients in the unfavourable group, whose tumours tended to be characterised by ER/PR negativity, expression of p53 and overexpression of HER2/neu.

### Prognostic indicators of outcome in 168 patients with high-risk breast cancer

As shown in Table 1, the characteristics of the entire cohort of 114 patients treated with HDCT and the 54 patients in the standard treatment group were similar, although a slightly higher percentage of HDCT-treated patients received standard doxorubicin containing adjuvant therapy and presented with low to intermediate grade tumours. When evaluating the entire group of 168 patients with HRBC, RFS was adversely affected by presentation with stage IIIB inflammatory features (RR, 1.74; 95% CI, 1.11–2.72; *P*=0.01), and for patients with positive post-mastectomy margins (RR, 1.94; 95% CI, 1.04–3.65; *P*=0.03); OS was shorter in patients with inflammatory disease (RR, 2.17; 95% CI, 1.35–3.48; *P*=0.001), positive margins (RR, 2.37; 95% CI, 1.25–4.51; *P*=0.01), and following standard adjuvant chemotherapy with a non-doxorubicin containing regimen (RR, 2.08; 95% CI, 1.04–4.16; *P*=0.04). Administration of adjuvant tamoxifen was associated with lower risk of death (RR, 0.65; 95% CI, 0.41–1.01; *P*=0.052).

**Figure 2:**
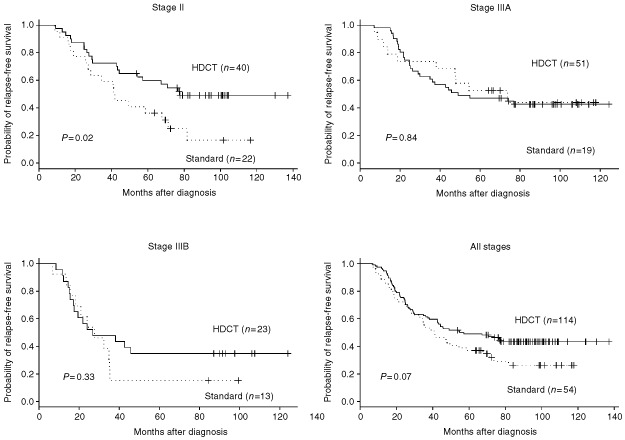
Relapse-free survival for stage II, IIIA and IIIB high-risk breast cancer patients treated with HDCT (solid lines) *vs* standard adjuvant therapy (dotted lines).

Seven-year projected RFS for stage II, IIIA and IIIB disease are 49% (95% CI, 36–68%), 43% (95% CI, 31–59%), and 35% (95% CI, 20–61%) following HDCT, *vs* 17% (95% CI, 5–50%), 44% (95% CI, 25–77%), and 15% (95 CI, 4–55%) with standard adjuvant therapy. The projected 7-year RFS for patients treated with HDCT for all stages is 43% (95% CI, 35–54%) *vs* 26% (95% CI, 16–43%) for the 54 patients treated with standard adjuvant therapy, as shown in Figure 2

**Figure 3:**
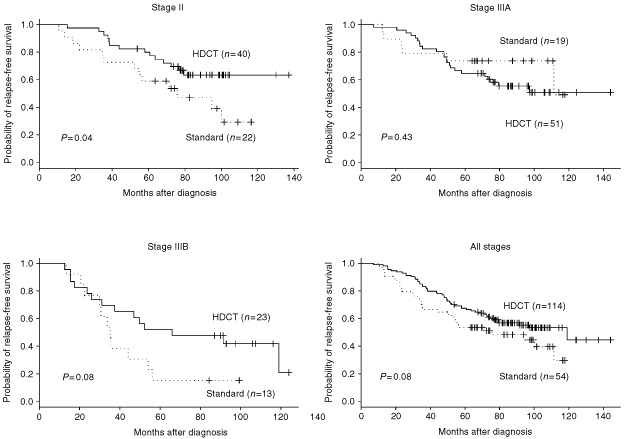
Overall survival for stage II, IIIA and IIIB high-risk breast cancer patients treated with HDCT (solid lines) *vs* standard adjuvant therapy (dotted lines).

. The projected 7-year OS for the entire group of patients treated with HDCT is 57% (95% CI, 48–67%) and 48% (95% CI, 36–65%) for the 54 patients treated with standard adjuvant therapy; OS for stage II, IIIA and IIIB disease is 64% (95% CI, 50–81%), 56% (95% CI, 43–72%), and 48% (95% CI, 31–73%) after HDCT *vs* 47% (95% CI, 29–76%), 74% (95% CI, 56–96%), and 15% (95 CI, 4–55%) after standard therapy as depicted in Figure 3

.

A trend toward lower risk of death (RR 0.66; 95% CI, 0.42–1.04; *P*=0.08) and relapse (RR 0.69; 95% CI, 0.46–1.03; *P*=0.07) was observed in the 114 patients treated with HDCT compared to the 54 ‘standard’ patients. A similar analysis restricted to those patients who received doxorubicin-based adjuvant therapy either prior to HDCT (109 patients), or as their only adjuvant treatment (45 patients), revealed improved outcome with HDCT for both OS (RR 0.61; 95% CI, 0.38–0.99; *P*=0.05) and RFS (RR 0.63; 95% CI, 0.41–0.95; *P*=0.03; data not shown). When assessing the role of HDCT, there was no significant difference between patients treated with either CAVP or CCVP.

## DISCUSSION

In addition to tumour size, receptor status, inflammatory features, proliferative rate, and grade, HER2/neu overexpression and expression of p53 protein have recently been identified as prognostic indicators in the treatment of standard risk breast (Muss *et al*, 1994; Paik *et al*, 1998; Thor *et al*, 1998; Paik *et al*, 2000). However, relatively few studies of predictive factors in the setting of HDCT for HRBC have been published. Candidates for HDCT constitute over 10% of all newly diagnosed cases of primary breast cancer; (Wood *et al*, 1994; Bonadonna *et al*, 1995) in our estimate, the 114 patients we treated with HDCT between 1989 and 1994 represented 0.5% of the potentially eligible patient population in Southern California, our primary service area (Somlo *et al*, 1997). Hence, our observations are deduced from a selected patient population.

We previously identified PR negativity as an independent predictor for relapse (Somlo *et al*, 1997). Nieto *et al* (1999) generated a predictive model based on tumour size, receptor status, and axillary nodal ratio; later they found HER2/neu overexpression (using monoclonal CB11 antibody-staining) as an additional independent predictor of RFS and OS primarily in an otherwise ‘favourable’ group of patients (Nieto *et al*, 2000). Using a polyclonal antibody-directed immunohistochemical stain the incidence of HER2/neu positive staining in our HRBC population was very similar to that reported in patients with stage II breast cancer (Thor *et al*, 1998) and to the selected, high risk population reported by Bitran *et al* (1996), although slightly lower then in the series by Nieto *et al* (1999). Since there is wide variation in the type and specificity of available antibodies and retrieving techniques, and the clinical significance of utilizing one antibody *vs* another is unclear (Ravdin *et al*, 1998), the predictive value of HER-2/neu amplification for choosing the optimal adjuvant therapy needs further validation.

We defined several factors which may, either independently or in concert, mark cancers with a high proliferation rate. Hence, it is not surprising that in univariate analysis the presence of p53 protein, overexpression of HER2/neu, and markers of high mitotic index, or de-differentiation (such as lack of expression of ER/PR) are all associated with poor outcome. Of the prognostic indicators tested, the persistence of tumour in the surgical margin, another adverse feature, may also be considered as an indirect measure of size and invasiveness. The established benefit associated with doxorubicin as standard adjuvant therapy is in agreement with the findings of larger studies in standard risk breast cancer (Bonadonna *et al*, 1995).

In keeping with our earlier observation, progesterone receptor status remained a predictor of RFS. However, multivariate Cox regression analysis may be of limited use when determining the biological role of individual markers due to their potential similarity as multiple measures of tumour aggressiveness. For example, since ER and PR staining and intensity are highly correlated, a model may choose only one feature, i.e., PR intensity. Similarly, the adverse predictive value associated with the presence of p53 expression may mask the significance of other markers of proliferation, such as Ki67. Grade, mitotic rate, and receptor status remain important characteristics of a predictive model for patients with HRBC. Even relatively nonspecific, immunohistochemically derived information on the degree of p53 expression and HER2/neu amplification may add to our ability to predict outcome. However, technical standardisation is needed for a more accurate determination of expression/amplification of these two later molecular markers.

Positive margin, inflammatory features, and lack of doxorubicin in the standard adjuvant regimen were confirmed to be adverse predictors of outcome for the entire cohort of 168 high-risk patients as well as for the 90 patient subset; however, instead of receptor-positivity for the tumour, tamoxifen therapy in the post-chemotherapy setting became a favourable predictor of outcome. Since only patients with ER and/or PR positive tumours received tamoxifen, this substitute finding points out the potential methodological problems when parameters with overlapping functions and features are evaluated.

Treatment of patients with HRBC continues to be a challenge, especially, since the recent attempts incorporating newer agents such as taxol into the standard adjuvant regimens have proven somewhat disappointing (Eifel *et al*, 2001). The number of patients treated with HDCT has dramatically decreased from its peak (Antman *et al*, 1997). Early expectations of a 25–30% survival benefit with HDCT over standard chemotherapy in patients with high-risk breast cancer were overstated partly based on data generated by fraud (Weiss *et al*, 2000). Two small and underpowered randomised studies could not confirm the benefit of adjuvant HDCT due, in part, to unrealistic (>25%) expectations of benefit (Rodenhuis *et al*, 1998; Hortobagyi *et al*, 2000). Data presented from a phase III US study has suggested a decreased relapse rate, but no effect on OS, because of unacceptably high treatment-related mortality following HDCT (Peters *et al*, 2001).

Preliminary data from a phase III Dutch trial in patients with ⩾4 involved axillary lymph nodes have suggested a 15% RFS and 10% OS benefit in the first 284 patients treated with a combination of carboplatin/thiotepa, and cyclophosphamide STAMP-V HDCT at 3-years, although these results have not yet been confirmed in all 885 study participants (Rodenhuis *et al*, 2000). In a prospective, randomised French trial of standard *vs* HDCT, RFS of 55% *vs* 71% was reported, favouring the HDCT arm at 3-years (Roche *et al*, 2001).

While one awaits maturation of data from these studies, as well as the two other, completed randomised trials from the United States, further clarification of predictors of poor risk is appropriate. Better selection of patients for studies of future HDCT programmes, as well as other investigational approaches for women with HRBC, may be possible after validation of this and other (Nieto *et al*, 1999) prognostic models.
